# The Usage of Concomitant Beta-Blockers with Vasopressors and Inotropes in Cardiogenic Shock

**DOI:** 10.3390/medsci10040064

**Published:** 2022-11-17

**Authors:** Rachel Ryu, Christopher Hauschild, Khaled Bahjri, Huyentran Tran

**Affiliations:** 1 College of Pharmacy, Western University of Health Sciences, 309 E. Second Street, Pomona, CA 91766, USA; 2 Loma Linda University Medical Center, 11223 Campus Street, Loma Linda, CA 92350, USA; 3 School of Pharmacy, Loma Linda University, 24745 Stewart Street, Loma Linda, CA 92350, USA

**Keywords:** cardiology, heart failure, inotropes, vasopressors, cardiogenic shock

## Abstract

Vasopressors and inotropes (Vs/Is) are widely used in the treatment of cardiogenic shock (CS). Despite improvements in hemodynamic variables and end-organ perfusion, these agents have been associated with an increase in mortality, potentially due to the increased risk of tachyarrhythmias—which we hypothesize may be mitigated by beta-blockers (BBs). We conducted a retrospective chart review of patients who received a V/I (dobutamine, milrinone, dopamine, and norepinephrine) for CS. The primary objective was to assess the effect of BB in patients receiving Vs/Is for CS. In our final analysis of 227 patients, those in the BB group were younger, were more likely to have acute coronary syndrome as the reason for admission, had more reduced left ventricular ejection fraction, were more likely to have coronary artery disease and atrial fibrillation as pre-existing co-morbidities, and had a lower rate of in-hospital mortality. Nevertheless, in our multivariable logistic regression analysis, concurrent BB usage with a V/I was not associated with a reduction in in-hospital mortality. Our present study sheds light on the importance and urgency of large, carefully designed clinical studies to optimize inpatient medical therapy, particularly evaluating the combination of V/I and BB, in this high-risk patient population.

## 1. Introduction

Cardiogenic shock (CS) is defined as a state of low cardiac output, leading to fatal hypoperfusion of the end organs [1,2,3]. The most common causes of CS are acute myocardial infarction and left ventricular dysfunction. Historically, mortality rates for CS have been up to 90%, but recently, the rate has improved significantly to 27% to 51% with the use of revascularization strategies—such as percutaneous coronary intervention in myocardial infarction-associated CS [2]. Mechanical devices, such as the intra-aortic balloon pump, have been used with unproven survival benefit. Fibrinolytic agents are effective for ST-segment elevation myocardial infarction, but mortality benefits have not been shown upon conversion to CS [1,2,3].

Limited evidence exists for the medical management of CS. The main goal of therapy is restoration of end-organ blood flow and tissue perfusion [1,2,3]. Vasopressors and inotropes (Vs/Is) can improve the hemodynamic profile of patients, although there is a risk for increased myocardial oxygen consumption, tachyarrhythmias, lactic acidosis, and possibly mortality. Thus, we hypothesize that the risks of Vs/Is may potentially be mitigated with the use of beta-blockers (BBs)—particularly by minimizing tachycardia and arrhythmias, and potentially mortality. The primary objective of our study was to assess the effect of BBs on in-hospital mortality in patients receiving Vs/Is for CS.

## 2. Materials and Methods

We conducted a retrospective chart review of all adult patients (age ≥ 18 years) who received a V/I (dobutamine, milrinone, dopamine, and norepinephrine) for CS between January 2017 and December 2018 in the coronary critical care unit at the Loma Linda University Medical Center. The patients included in this study were primarily derived from our previous study, in which we assessed predictors of in-hospital mortality in patients with CS requiring a V/I [4]. CS was classified with the International Statistical Classification of Diseases (ICD) code-10 R57.0 or ICD-9 785.51. Patients were excluded if they were not on a cardiology service, had other indications for a V/I (e.g., sepsis/septic shock), were on mechanical circulatory support, or were on V/I as continuation of home inotropic support.

In this study, we focused on the effects of BBs. As a result, patients were divided into two groups: BB group vs. no BB group. Baseline BB use was defined as the presence of a BB order within 48 h prior to the initiation of a V/I. Using a standardized form, data collection was conducted through review of the electronic medical record, and variables included the following: demographic characteristics (gender, race, and age), reason for admission, heart failure etiology, baseline left ventricular ejection fraction, baseline labs, co-morbid conditions, hospital length of stay, in-hospital mortality, and status of orthotopic heart transplantation in surviving patients.

In-hospital mortality status was defined as death from all causes during the hospitalization. Hemodynamic variables, including heart rate (HR) in beats per minute (bpm) and blood pressure (BP) in mmHg, were collected at the time of V/I initiation, at maximum V/I doses, and at discharge or death. All V/I agents, doses, and duration were collected. Descriptive statistics in the form of mean and standard deviation were used for continuous variables, and numbers and percentages for categorical variables. The statistical significance of differences was compared using the chi-squared test for categorical variables, and two-sample t-test for continuous variables. All tests were two-tailed with a significance level of *p* < 0.05. Significant variables from the bivariate analysis were included in the multivariable logistic regression to assess the effect on mortality. Multivariable analysis was conducted using logistic regression with the backward stepwise method, and the odds ratio and 95% confidence intervals were estimated. SPSS version 26.0 was used to analyze the data.

## 3. Results

Of 227 patients in our final analysis, the average age was 65.4 +/− 15.1 years, 66.1% were male, and 41% were Hispanic (Table 1). The most common reason for hospital admission was acute decompensated heart failure (47.8%), followed by acute coronary syndrome (34.4%). Patients primarily had ischemic heart failure (53.3%) and reduced left ventricular ejection fraction (mean 26 +/− 14.9%). The majority of patients had elevated serum creatinine, liver function tests, and serum lactate. The most common pre-existing co-morbidity was hypertension (67.4%). Patients stayed in the hospital for an average of 8.4 +/− 9.6 days. The in-hospital mortality rate was found to be 18.5%, and of surviving patients (*n* = 185), 2.7% received an orthotopic heart transplantation during the hospitalization.

Overall, patients in the BB group compared with the no BB group were younger (61.7 +/− 13.2 vs. 68.6 +/− 12.1 years; *p* < 0.001), were more likely to have acute coronary syndrome as the reason for admission (44.8% vs. 15.5%; *p* < 0.001), had more reduced left ventricular ejection fraction (22 +/− 15.7% vs. 31 +/− 17.9%; *p* < 0.001), had more coronary artery disease (71.3% vs. 33.3%; *p* < 0.001), had more atrial fibrillation (44.8% vs. 31%; *p* = 0.04), and had a lower rate of in-hospital mortality (6.3% vs. 39.3%; *p* < 0.001) (Table 1). With respect to hemodynamic variables between the two groups, patients receiving BBs had a significantly higher HR when V/I was started, lower HR when V/I was at maximum doses, and lower systolic and diastolic BP at maximum V/I doses (Table 2).

A multivariable logistic regression was performed to assess the adjusted impact of BB use on in-hospital mortality (Table 3). Other significant covariates in the analysis included age ≥ 65 years, acute decompensated heart failure as the reason for admission, acute coronary syndrome as the reason for admission, left ventricular ejection fraction on admission ≤ 25%, coronary artery disease as a co-morbid condition, atrial fibrillation as a co-morbid condition, HR when V/I started ≤85 bpm, HR on maximum V/I ≥95 bpm, and BB use. In this model, none of the variables were independent risk factors for in-hospital mortality. In our multivariable analysis, concomitant BB use with V/I was not associated with a reduction in in-hospital mortality.

## 4. Discussion

In our study, we compared patients receiving BBs within 48 h prior to the initiation of a V/I, to those without such BB therapy, in patients receiving Vs/Is for CS. When comparing hemodynamic variables between the groups, it was found that patients in the BB group had less tachycardia and hypertension while on V/I therapy. Although the in-hospital mortality rate was significantly lower in patients in the BB group, we found that concomitant BB usage with V/I was not associated with a reduction in in-hospital mortality based on our multivariable regression analysis.

In a recent subgroup analysis of the DOREMI trial, patients receiving BBs 24 h prior to undergoing CS were compared with those not receiving such BB treatment [5]. The BB group was found to have fewer episodes of cardiac arrest and lower mortality in the early resuscitative period of CS. However, these benefits were not sustained throughout the hospitalization and there was no difference in mortality at the time of discharge. The authors suggested a paradoxically protective effect of BB in the early CS period. In a separate study of septic myocardial depression and severe sepsis, concurrent BB with phosphodiesterase inhibitors, such as milrinone, showed an improved control of HR while preserving cardiac index, as well as an increase in 28-day overall survival [6,7]. BBs, particularly carvedilol and metoprolol, may attenuate the hemodynamic response desired by beta-adrenergic inotropes, but not phosphodiesterase inhibitors such as enoximone, in patients with chronic heart failure [8].

Vs/Is may increase myocardial oxygen demand and ischemic burden, and predispose patients to malignant arrhythmias, sinus tachycardia, increased ventricular rate in patients with atrial fibrillation, and catheter-related infections [1,2,3]. As a result, international societies suggest that Vs/Is should be reserved for severe/refractory heart failure, or CS with hemodynamic instability [1,2,3,9]. If a V/I is to be used, the consensus is to use the lowest doses for the shortest duration to prevent adverse effects. Once V/I treatment is initiated, it is recommended that routine assessment be performed to evaluate the appropriateness of discontinuation.

BBs are one of the first-line drug classes recommended to improve morbidity and mortality in patients with chronic heart failure, particularly those with reduced ejection fraction [1,3]. It is known that BBs are contraindicated in the setting of overt heart failure or low-output states due to the negative inotropic effects of BBs. However, the consensus is to continue BB treatment in patients who were previously on them, during an acute heart failure hospitalization. Dose reduction or the discontinuation of BBs in this setting has shown to cause poor outcomes [10].

In the setting of acute decompensated heart failure requiring inotropic support, BBs have shown to reduce the rate of V/I-induced ventricular arrhythmias, lower rates of premature ventricular contractions, ventricular couplets, and total ventricular arrhythmias [11,12]. In a randomized, double-blind, multi-center study by Böhm and colleagues, the investigators showed that in patients with acute heart failure requiring inotropes, BBs on admission and at discharge led to lower 31-day mortality, and thus recommended continuation while in the hospital [13]. In a separate retrospective single-center chart review study, Delmas and colleagues showed that in patients with CS, BBs on admission led to lower long-term mortality in patients, although it was not specified whether BBs were continued throughout the hospitalization [14].

In conclusion, the benefits of BBs in chronic reduced ejection fraction heart failure are well established, and there is strong agreement to continue BB treatment while patients are undergoing an acute heart failure episode. In the case of overt heart failure or CS without V/I support, the usage of BBs is not recommended. Nevertheless, in the setting of CS with anticipated side effects of tachycardia and arrhythmias due to Vs/Is, the impact of the concurrent usage of BBs with Vs/Is is unclear. In our multivariable analysis, concomitant BB use with a V/I was not associated with a decrease in in-hospital mortality in CS patients.

Our study had limitations, which include those inherent to the observational, retrospective nature of study, such as possible selection or confounding bias. This was a descriptive study and therefore did not have a control arm; thus, only associations, not causations, can be determined. Given our small sample size, the probability of making a type II statistical error is increased and our study may lack the statistical power to detect small effects. For clinical data, we were unable to account for the exact timing of CS resolution due to the ambiguous, multifactorial nature of the disease progression. Euvolemia may or may not have occurred in patients, and even so, the precise timing was unclear. BB and V/I were each analyzed as a drug class, which may not capture the receptor selectivity and potency of each individual drug. Lastly, our study excluded patients on mechanical circulatory support; thus, the patients in our sample may have had less severe disease at baseline.

## 5. Conclusions

Overall, the role of BBs is well-established in chronic heart failure, especially those with reduced ejection fraction. Furthermore, it is generally advised to continue BBs in the hospital during an acute heart failure episode and discouraged in overt heart failure or CS without V/I support. Nevertheless, in the setting of CS with anticipated side effects of tachycardia and arrhythmias due to V/I, the impact of concurrent usage of BBs with Vs/Is is unclear. In our multivariable analysis, concomitant BB use with a V/I was not associated with a decrease in in-hospital mortality in CS patients.

Large, randomized, controlled studies are warranted to further characterize the role of concomitant BB use in CS patients receiving a V/I, and the effect on mortality. Our findings should be hypothesis-generating and aid in the development of future studies. Our present study highlights the need for well-designed studies to identify the risks associated with mortality in CS, further characterize the disease state and pathogenesis, and develop ways to improve patient outcomes by way of optimizing treatment and mitigating adverse effects of therapy.

## Figures and Tables

**Table 1:** Baseline characteristics for entire cohort, and separated by BB group vs. no BB group.

Variable		N (%) or Mean ± SD			*p*-Value
		Total (*n* = 224)	BB Group (*n* = 143)	No BB Group (*n* = 84)	
Age on Admission (years)		65.4 ± 15.1	61.7 ± 13.2	68.6 ± 12.1	<0.001 *
Gender	Males	150 (66.1%)	94 (65.7%)	56 (66.7%)	0.886
	Females	77 (33.9%)	49 (34.3%)	28 (33.3%)	0.886
Race/Ethnicity	Hispanic	93 (41.0%)	56 (39.2%)	32 (38.1%)	0.873
	White	82 (36.1%)	51 (35.7%)	31 (36.9%)	0.849
	Black	28 (12.3%)	14 (9.8%)	9 (10.7%)	0.826
	Other	24 (10.6%)	22 (15.4%)	12 (14.3%)	0.826
Reason for Admission	ADHF	107 (47.8%)	67 (46.9%)	40 (47.6%)	0.912
	ACS	77 (34.4%)	64 (44.8%)	13 (15.5%)	<0.001 *
	Other	40 (17.9%)	21 (14.7%)	19 (22.6%)	0.131
Heart Failure Etiology	Ischemic	121 (53.3%)	75 (52.4%)	46 (54.8%)	0.738
	Non-Ischemic	89 (39.2%)	55 (38.5%)	34 (40.5%)	0.764
	Combined	17 (7.5%)	13 (9.1%)	4 (4.8%)	0.23
LVEF (%) on Admission		26 ± 14.9	22 ± 15.7	31 ± 17.9	<0.001 *
Labs on Admission	SCr (mg/dL)	1.7 ± 0.7	1.6 ± 1.5	1.8 ± 1.9	0.411
	AST (units/L)	88.9 ± 42.7	87.2 ± 44.8	88.4 ± 38.9	0.832
	ALT (units/L)	90.1 ± 45.6	89.1 ± 45.6	91.2 ± 59.8	0.782
	T. bili (mg/dL)	2.1 ± 0.4	2.1 ± 8.5	2.2 ± 9.4	0.936
	Serum Lactate (mmol/L)	6.0 ± 3.2	5.9 ± 2.2	6.1 ± 3.1	0.604
Co-morbid Conditions	Hypertension	153 (67.4%)	96 (67.1%)	57 (67.9%)	0.911
	CAD	130 (57.3%)	102 (71.3%)	28 (33.3%)	<0.001 *
	Diabetes Mellitus	107 (47.1%)	64 (44.8%)	43 (51.2%)	0.347
	AF	90 (39.6%)	64 (44.8%)	26 (31%)	0.04 *
	Valvular Disease	72 (31.7%)	44 (30.8%)	28 (33.3%)	0.689
	Asthma/COPD	42 (18.9%)	27 (18.9%)	15 (17.9%)	0.849
	History of VTE	31 (13.7%)	18 (12.6%)	13 (15.5%)	0.54
	Meth use (current/previous)	30 (13.3%)	15 (10.5%)	15 (17.9%)	0.114
	OSA	17 (7.5%)	11 (7.7%)	6 (7.1%)	0.881
	ESRD	10 (4.4%)	4 (2.8%)	6 (7.1%)	0.124
Hospital Length of Stay (days)		8.4 ± 9.6	8.5 ± 6.6	8.4 ± 5.9	0.906
In-hospital Mortality		42 (18.5%)	9 (6.3%)	33 (39.3%)	<0.001 *
OHT among Survivors (*n* = 185)		5 (2.7%)	3 (2.1%)	2 (2.4%)	0.889

* Significant at an alpha value of 0.05. Abbreviations: acute decompensated heart failure (ADHF), acute coronary syndrome (ACS), alanine transferase (ALT), aspartate transaminase (AST), atrial fibrillation (AF), beta-blockers (BBs), chronic obstructive pulmonary disease (COPD), coronary artery disease (CAD), end-stage renal disease (ESRD), left ventricular ejection fraction (LVEF), methamphetamine (meth), obstructive sleep apnea (OSA), orthotopic heart transplantation (OHT), serum creatinine (SCr), standard deviation (SD), total bilirubin (T. bili), venous thromboembolism (VTE).

**Table 2:** Comparison of hemodynamic variables between BB group and no BB group.

Variable	Mean ± SD		*p*-Value
	BB Group (*n* = 143)	No BB Group (*n* = 84)	
HR when V/I started (bpm)	90.04 ± 18.49	82.11 ± 18.72	0.002 *
Systolic BP when V/I started (mmHg)	84.01 ± 18.52	82.2 ± 19.29	0.49
Diastolic BP when V/I started (mmHg)	54.32 ± 17.23	52.14 ± 13.16	0.285
HR on maximum V/I (bpm)	90.15 ± 20.11	98.40 ± 18.32	0.002 *
Systolic BP on maximum V/I (mmHg)	99.33 ± 19.43	110.82 ± 19.79	<0.001 *
Diastolic BP on maximum V/I (mmHg)	64.94 ± 15.92	69.41 ± 15.15	0.041 *
HR on discharge/death (bpm)	62.14 ± 28.84	59.23 ± 32.14	0.495
Systolic BP on discharge/death (mmHg)	78.38 ± 32.48	74.89 ± 52.11	0.58
Diastolic BP on discharge/death (mmHg)	45.93 ± 21.88	47.61 ± 33.01	0.678

* Significant at an alpha value of 0.05. Abbreviations: beats per minute (bpm), blood pressure (BP), heart rate (HR), standard deviation (SD), vasopressor or inotrope (V/I).

**Table 3:** Multivariable logistic regression of predictors for in-hospitality mortality.

Risk Factor	OR (95% CI)	*p*-Value
Age ≥ 65 years	2.32 (0.82–3.38)	0.056
Reason for admission: ADHF	1.89 (0.87–2.24)	0.068
Reason for admission: ACS	1.42 (0.94–2.43)	0.051
LVEF on admission ≤25%	1.81 (0.72–3.4)	0.065
Co-morbid condition: CAD	1.5 (0.97–2.12)	0.068
Co-morbid condition: AF	1.25 (0.7–2.1)	0.432
HR when V/I started ≤85 bpm	5.23 (0.98–11.21)	0.871
HR on maximum V/I ≥95 bpm	4.87 (0.87–8.14)	0.951
BB use	0.98 (0.88–1.09)	0.052

Abbreviations: acute coronary syndrome (ACS), acute decompensated heart failure (ADHF), atrial fibrillation (AF), beats per minute (bpm), beta-blocker (BB), blood pressure (BP), confidence interval (CI), coronary artery disease (CAD), heart rate (HR), left ventricular ejection fraction (LVEF), odds ratio (OR), vasopressor or inotrope (V/I).

## Data Availability

De-identified participant data, study methodology, and statistical analyses will be shared upon reasonable request by the corresponding author.
